# A 2-Month Follow-Up Study of Psychological Distress among Italian People during the COVID-19 Lockdown

**DOI:** 10.3390/ijerph17218180

**Published:** 2020-11-05

**Authors:** Paolo Roma, Merylin Monaro, Marco Colasanti, Eleonora Ricci, Silvia Biondi, Alberto Di Domenico, Maria Cristina Verrocchio, Christian Napoli, Stefano Ferracuti, Cristina Mazza

**Affiliations:** 1 Department of Human Neuroscience, Sapienza University of Rome, 00185 Rome, Italy; paolo.roma@uniroma1.it (P.R.); marco.colasanti@hotmail.com (M.C.); silviabiondi14@gmail.com (S.B.); stefano.ferracuti@uniroma1.it (S.F.); 2 Department of General Psychology, University of Padova, 35131 Padova, Italy; merylin.monaro@unipd.it; 3 Department of Neuroscience, Imaging and Clinical Sciences, “G. d’Annunzio” University of Chieti-Pescara, 66100 Chieti, Italy; eleonoraricci25@gmail.com; 4 Department of Psychological, Health and Territorial Sciences, University “G. d’Annunzio” of Chieti-Pescara, 66100 Chieti, Italy; alberto.didomenico@unich.it (A.D.D.); mc.verrocchio@unich.it (M.C.V.); 5 Department of Medical Surgical Science and Translational Medicine, Sapienza University of Rome, 00189 Rome, Italy; christian.napoli@uniroma1.it

**Keywords:** COVID-19, lockdown, follow-up, Italian citizens, depression, anxiety, stress, mental health intervention

## Abstract

The spread of coronavirus disease 2019 (COVID-19) has called for unprecedented measures, including a national lockdown in Italy. The present study aimed at identifying psychological changes (e.g., changes in depression, stress, and anxiety levels) among the Italian public during the lockdown period, in addition to factors associated with these changes. An online follow-up survey was administered to 439 participants (original sample = 2766), between 28 April and 3 May 2020. A paired sample t-test tested for differences in stress, anxiety, and depression over the period. Multivariate regression models examined associations between sociodemographic variables, personality traits, coping strategies, depression, and stress. Results showed an increase in stress and depression over the lockdown, but not anxiety. Negative affect and detachment were associated with higher levels of depression and stress. Higher levels of depression at the start of the lockdown, as well as fewer coping strategies and childlessness, were associated with increased depression at follow-up, whereas higher levels of stress at the start of the lockdown and younger age were associated with higher stress at follow-up. These results may help us to identify persons at greater risk of suffering from psychological distress as a result lockdown conditions, and inform psychological interventions targeting post-traumatic symptoms.

## 1. Introduction

The detrimental effects of a prolonged lockdown period on mental health are widely documented in the literature, which reports an increase in depression, anxiety, post-traumatic stress symptoms, low mood, irritability, stress, and sleep-related disturbances under lockdown conditions [1,2,3,4]. Therefore, it is of paramount importance to monitor how the recent lockdown relating to COVID-19 has affected psychological distress levels in the general population.

Longitudinal studies investigating the psychological effects of the COVID-19 lockdown are scarce: Wang et al. [5], for example, surveyed the general population in China during the initial outbreak and conducted a 1-month follow-up during the peak of the epidemic; surprisingly, the authors found no significant changes in levels of stress, anxiety, and depression, as measured with the Depression, Anxiety and Stress Scale–21 (DASS-21). This result is not consistent with the findings of similar studies reported in the literature. There are two potential reasons for this: first, for reasons of anonymity, the populations differed between the initial and the follow-up survey; and second, responses to both surveys were collected from residents of various Chinese cities that faced different situations in terms of the spread of COVID-19 and associated lockdown measures. Another study, carried out by Planchuelo-Gómez et al. [6], assessed the temporal evolution of the psychological effects of the COVID-19 lockdown in Spain, between the end of March and the end of April 2020. The authors found significantly higher scores in their follow-up survey for all dimensions of psychological distress (i.e., anxiety, stress, depression), as assessed by the DASS-21. Finally, a recent longitudinal probability sample survey of the UK population [7] found an increase in psychological distress compared to previous years, especially among women, younger age groups, and individuals living with children.

One of the first survey studies of psychological distress in Italy during the initial phase of the COVID-19 outbreak [8] identified several factors associated with increased anxiety, depression, and stress. Using the aforementioned research as a starting point, the aim of this paper was to conduct a follow-up survey to gather further insight into the impact of the COVID-19 lockdown on the mental health of the Italian public. In the follow-up study, the focus was exclusively on factors that had previously been found to be significantly associated with increased psychological distress, across any dimension (i.e., anxiety, depression, stress), as measured by the Italian version of the DASS-21 [9]. Specifically, three factors had been shown to be associated with an increase in all dimensions: female gender and the personality domains of negative affect (i.e., a tendency to experience unpleasant feelings such as anger or anxiety and labile emotionality) and detachment (i.e., depressive affect and interpersonal withdrawal), as measured by the Personality Inventory for the Diagnostic and Statistical Manual of Mental Disorders (DSM-5–Brief Form, PID-5-B) [10]. Moreover, the following factors had been found to be associated with increased anxiety: a history of stressful situations, a history of medical issues, and an infected relative. Furthermore, the following factors had been found to be associated with increased depression: a history of stressful situations, a history of medical issues, an infected acquaintance, and not having children. Finally, an association between young age and a higher level of stress was found.

In addition, the association between resilient coping and psychological distress during the lockdown was also assessed in this study. Resilient coping can be defined as “*a tendency to effectively use cognitive appraisal skills in a flexible, committed approach to active problem solving despite stressful circumstances*” ([11], p.95). Research has indicated that adaptive coping responses are associated with better physical and mental health in individuals facing traumatic events or health-related stressors [12,13,14,15]. Furthermore, studies conducted during past epidemics (e.g., SARS) and the current COVID-19 pandemic have highlighted that maladaptive coping strategies can play a role in the development of psychological problems, whereas adaptive coping strategies are negatively associated with psychological illness [16,17].

In the present follow-up study, we aimed at testing changes in depression, stress, and anxiety levels in Italian people over the 2-month lockdown period. In line with previous studies, we hypothesized that psychological distress would have increased. We also sought to identify which factors were strongly associated with higher levels of distress. The association between the COVID-19 pandemic and significant levels of psychological distress in the general population is well documented. However, analysis of the longitudinal evolution of anxiety, depression, and stress levels via a follow-up study could help us determine whether this increase in distress is sustained, reduced, or exacerbated. Furthermore, research of this nature could provide us with further insight into the mental health of the Italian population and the development of more targeted interventions.

## 2. Materials and Methods

### 2.1. Procedures

We employed an anonymous online questionnaire to trace the Italian public’s psychological response to the COVID-19 lockdown, 2 months after it began. The survey was administered cross-sectionally on the same online platform as initially employed [8]. The link was disseminated to participants who had consented in the initial survey to being contacted for follow-up, and who had provided their email address for this purpose. Data were collected over the last 6 days of the lockdown in Italy, from 28 April to 3 May 2020, using a survey comprised of both sociodemographic questions (regarding age, education, biological sex, and information related to COVID-19) and the DASS-21 scale (aimed at investigating participants’ mental health). We then supplemented this survey data with previously collected data on personality functioning (e.g., PID-5-BF Negative Affect and PID-5-BF Detachment) and coping strategies (e.g., BRCS) from the first survey, which was administered during the initial phase of the lockdown in Italy [8]. Expedited ethics approval was obtained from the Institutional Board of the Department of Human Neuroscience, Faculty of Medicine and Dentistry, “Sapienza” University of Rome (IRB-2020-6), in conformity with the principles embodied in the Declaration of Helsinki.

### 2.2. Participants

In the first national survey [8], 1518 respondents (out of a total sample of 2766) expressed their willingness to be contacted again for a follow-up study. All of these participants were invited via email to participate in the research, and 465 agreed to take part. Seventeen participants were excluded from the analysis because they reported an email address that did not match the one previously supplied; a further nine participants registered a double set of answers, from which only one set was retained. Thus, the final sample comprised 439 participants, with 110 males (25.1%) and 329 (74.9%) females. The average age was 34.70 years (*SD* = 13.15; range 18–70). More descriptive statistics are presented in Table 1. All participants voluntarily responded to the anonymous survey and indicated their informed consent within. The procedures were clearly explained, and participants could interrupt or quit the survey at any point without explaining their reasons for doing so.

### 2.3. Survey Measures

#### 2.3.1. Initial Measures

*Personality functioning*. Personality functioning was investigated using the Personality Inventory for the DSM-5—Brief Form—Adult (PID-5-BF) [10]. The PID-5-BF is a 25-item self-rated scale that assesses five personality trait domains: negative affect (e.g., *I worry about almost everything; I get emotional easily, often for a small reason*), detachment (e.g., *I often feel like nothing I do matters; I steer clear of romantic relationships*), antagonism (e.g., *I don’t like to get too intimate with people; I long for attention*), disinhibition (e.g., *People would describe me as reckless; I feel like I act completely on impulse*), and psychoticism (e.g., *I often have thoughts that make sense to me, but others say they’re weird; Often things around me seem unreal, or more real than usual*). Each domain is measured through five items that are rated on a 4-point Likert scale ranging from *0* (*very false or often false*) to 3 (*very true or often true*). The overall measure generates scores in the range of 0–75, with higher scores indicating greater overall personality dysfunction. Each trait domain receives a score in the range of 0–5, with higher scores indicating greater dysfunction in that specific personality trait domain. In the present study, the dimensions of negative affect and detachment were retained, because the literature indicates a relationship between these traits and internalizing psychopathology (e.g., depression and anxiety) [10,18,19].

*Coping strategies*. The Brief Resilient Coping Scale (BRCS) [11] is a 4-item questionnaire that is designed to capture the tendency to cope with stress in a highly adaptive manner (e.g., *During lockdown, I look for creative ways to alter difficult situations; During lockdown, I actively look for ways to replace the losses I encounter in life*). It is rated on a 5-point Likert scale ranging from 1 (*does not describe me at all*) to 5 (*describes me very well*). Total scores range from 4–20, with scores of 4–13 indicating low resilience coping, 14–16 indicating medium resilience coping, and 17–20 indicating high resilience coping. In the present sample, the BRCS showed a Cronbach’s alpha of 0.74.

#### 2.3.2. Follow-Up Measures

*Sociodemographic data*. Sociodemographic data were collected with regards to biological sex, age, education, marital and parental status, employment status, residential location during the COVID-19 outbreak (e.g., *Region of residence at the time of the spread of COVID-19?; Did you move following the COVID-19 emergency?*), and any history of stressful situations and medical problems (e.g., *In the past year, have you experienced the following situations (e.g., death of a family member; dismissal)?; Do you have current or past medical problems?)*. Moreover, further information related to COVID-19 was collected. Specifically, participants were asked if they were following the government advice to stay at home or if they were continuing to go to work (e.g., *Are you currently in a condition where you have to go out for work?*). They were also asked if any acquaintances or loved ones were (or had been) infected with COVID-19 (e.g., *Have you had acquaintances infected with COVID-19?*; *Have you had loved ones (e.g., family members, friends) infected with COVID-19*?).

*Psychological impact and mental health*. Mental health was measured using the Depression, Anxiety and Stress Scale—21 items (DASS-21) [7]. The DASS-21 is a set of three self-report scales designed to measure the emotional states of depression, anxiety, and stress. Each of the three scales contains seven items, divided into subscales with similar content. Items 3, 5, 10, 13, 16, 17, and 21 comprise the Depression subscale (e.g., *In the last 7 days, I couldn’t seem to experience any positive feeling at all*; *In the last 7 days, I found it difficult to work up the initiative to do things*); items 2, 4, 7, 9, 15, 19, and 20 comprise the Anxiety subscale (e.g., *In the last 7 days, I experienced trembling; In the last 7 days, I was worried about situations in which I might panic and make a fool of myself*); and items 1, 6, 8, 11, 12, 14, and 18 comprise the Stress subscale (e.g., *In the last 7 days, I tended to over-react to situations; In the last 7 days, I felt that I was using a lot of nervous energy*). All subscales are rated on a 4-point Likert scale ranging from *0* (*never*) to 3 (*almost always*). The DASS-21 obtained high reliabilities in the Italian validation study, with Cronbach’s alphas of 0.74, 0.82, and 0.85 for the Anxiety, Depression, and Stress subscales, respectively; Cronbach’s alpha for the total scales was 0.90. In our sample, Cronbach’s alphas were 0.90, 0.84 and 0.92 for the Depression, Anxiety, and Stress subscales, respectively. Cronbach’s alpha for the total scales was 0.95.

## 3. Statistical Analysis

Statistical analysis was performed using JASP 0.13.1 [20]. A paired sample t-test was run on DASS-21 subscale scores to measure differences in stress, anxiety, and depression levels between the initial and the final period of the lockdown. To address the problem of multiple testing, the Bonferroni correction was applied, dividing the *p*-value by the number of tested scales (*n* = 3) and setting the significance level to 0.017 [21].

Second, multivariable regression analyses were run to investigate the association between DASS-21 subscale scores during the final period of the lockdown and the following independent variables: BRCS score, DASS-21 subscale scores from the initial period of the lockdown, and factors previously found to be significant in the first period of the lockdown [8]. The collinearity assumption was checked prior to running the model. The analysis was performed using stepwise variable selection (with the threshold level of statistical significance for each variable to enter the model set to *p* < 0.05). The results were reported using unstandardized coefficients, as recommended by Friedrich [22].

## 4. Results

### 4.1. Differences Between the Initial and the Final Period of the Lockdown

**Figure 1:**
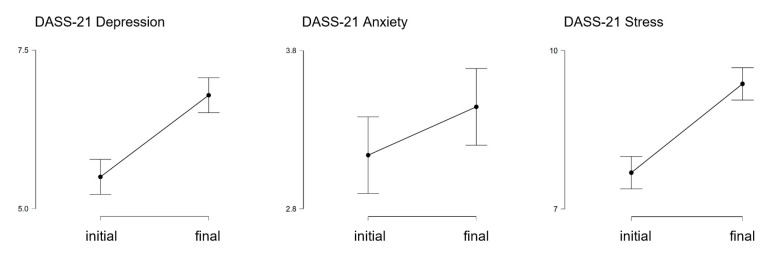
Descriptive plots comparing depression, anxiety, and stress levels, as measured with the DASS-21, between the initial and the final period of the lockdown.

Table 2 reports the average scores on the DASS-21 subscales between the initial and the final period of the lockdown. A graphical representation is provided in Figure 1. The results of the paired sample t-test revealed a difference in both the Depression and the Stress subscale scores (see Table 2).

As concerns depression, 53.30% of participants presented a higher score on the Depression subscale during the final period of the lockdown, while 46.70% showed a lower (31.21%) or a stable (15.49%) score. Similarly, 58.31% of participants scored higher on the Stress subscale, while 30.52% scored lower and 11.16% scored similarly. Conversely, no difference was found between the initial and the final period of the lockdown on the Anxiety subscale. As the *p*-value of the t-test analysis did not reach the significance level (*t* = −1.752, *p* = 0.080, *d* = −0.084, CI [−0.177, 0.010]), we did not consider this variable for further analysis (i.e., multiple regression).

### 4.2. Regression Analysis

A multiple regression analysis was run, including as predictors the demographic and psychological variables that prior research [4] has shown to have impacted depression and stress levels during the first period of the lockdown. The BRCS and DASS-21 subscale scores collected during the initial period of the lockdown were also included as predictors.

**Figure 2:**
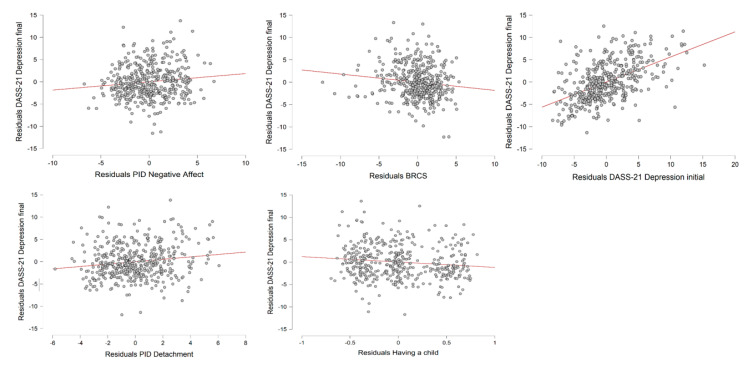
Partial regression plots representing the relationship between the DASS-21 Depression subscale score during the final period of the lockdown and the independent variables that accounted for a significant proportion of the variance according to the linear regression analysis.

As concerns depression, the Depression subscale score during the final period of the lockdown was set as the dependent variable, while gender, education level, working position, having a child, an infected acquaintance, a history of stressful situations, a history of medical problems, the PID-5-BF Negative Affect score, the PID-5-BF Detachment score, the BRCS score, and the DASS-21 Depression subscale score from the initial period of the lockdown were entered as covariates. The final model accounted for a significant proportion of the variance in level of depression (*R*^2^ = 0.519, Adjusted *R*^2^ = 0.514, *F*-change_(1,433)_ = 5.319, *p* = 0.022). Having a child and scores for the PID-5-BF Negative Affect, PID-5-BF Detachment, BRCS, and DASS-21 Depression subscale during the initial period of the lockdown were found to be significant predictors of level of depression during the final period of the lockdown (see Table 3). The relationship between the dependent variable and the statistically significant independent variables is graphically represented in Figure 2. Gender, education level, working position, an infected acquaintance, a history of stressful situations, and a history of medical problems were excluded from the model.

**Figure 3:**
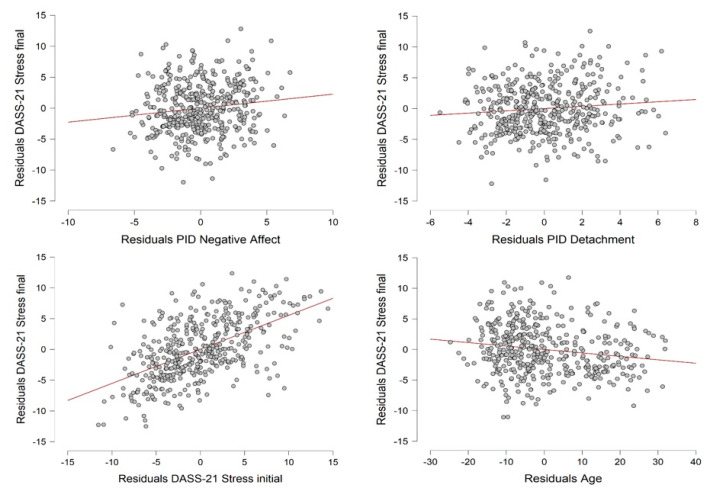
Partial regression plots representing the relationship between the DASS-21 Stress subscale score during the final period of the lockdown and the independent variables that accounted for a significant proportion of the variance according to the linear regression analysis.

Regarding stress, the DASS-21 Stress subscale score recorded in the final period of the lockdown was set as the dependent variable. The independent variables were gender, age, an infected family member, a history of stressful situations, a history of medical problems, the PID-5-BF Negative Affect score, the PID-5-BF Detachment score, the BRCS score, and the DASS-21 Stress subscale score during the initial period of the lockdown. The final model accounted for a significant proportion of the variance in level of stress (*R*^2^ = 0.494, Adjusted *R*^2^ = 0.489, *F*-change_(1,434)_ = 4.729, *p* = 0.030). Age and the PID-5-BF Negative Affect score, PID-5-BF Detachment score, and DASS-21 Stress subscale score during the initial period of the lockdown were included in the model (see Table 4). A graphical representation of the relationship between the dependent variable and the statistically significant independent variables is provided in Figure 3. Gender, an infected family member, a history of stressful situations, a history of medical problems, and the BRCS score were not significant predictors.

## 5. Discussion

The present report aimed at briefly documenting the evolution of psychological distress in the Italian public during the COVID-19 lockdown. We compared levels of stress, depression, and anxiety reported by 439 participants between the initial and the final period of the lockdown (i.e., a 2-month period). The results showed an increase in stress and depression levels, but no increase in anxiety.

Depression level at the initial phase of the lockdown was significantly associated with an increase in depression over the lockdown period. This result is not startling, as it indicates that individuals who presented higher levels of depression during the initial phase of the lockdown exhibited more depressive symptoms during the final period of the lockdown. In detail, we observed an average increase of 1.29 points on the DASS-21 Depression subscale at follow-up. Other longitudinal studies have yielded similar findings: for instance, Planchuelo-Gómez et al. [6] indicated an increase of 1.05 points on the DASS-21 Depression subscale over the study period, with an average score of 5.06 for the first survey and 6.11 for the follow-up.

Negative affect and detachment were also found to be associated with increased depression over the lockdown period. This result is aligned with the observations of both the original and other studies, indicating that these personality traits are good indices of internalizing psychopathology [18,19,23]. Accordingly, recent COVID-19 studies have reported that, during the lockdown, individuals with high levels of neuroticism experienced more negative affect in their daily lives [24], reported more concerns, and were more pessimistic about the duration estimates of the COVID-19 outbreak [25].

Our results also indicated that a more resilient coping style, as measured by the BRCS scale, was negatively associated with depression levels: individuals who engaged in active and flexible strategies under stressful circumstances were more likely to experience fewer depressive symptoms. Previous research has consistently indicated that active and flexible coping strategies are associated with lower psychological distress and depression [26]; similar results have also been found by studies of coping mechanisms during the COVID-19 pandemic see, e.g., [27,28]. Lastly, the present research found that childlessness increased levels of depression [8]. One interpretation of this could be that children are a source of positive emotions, and they may motivate parents to take initiative and have trust in the future; in this way, parents may derive personal value from the parental role. Furthermore, the complex task of childcare throughout the lockdown period may have protected parents against depressive feelings (or an awareness of these feelings), as measured by the DASS-21 (e.g., “I was unable to become enthusiastic about anything”); however, it may have nonetheless led parents to experience greater distress or exhaustion [29,30,31].

Finally, level of stress at the initial phase of the lockdown was found to be significantly associated with increased stress over the lockdown period: individuals who presented higher levels of stress during the initial phase of the lockdown exhibited higher levels of stress during the later phase. In detail, we observed an average increase of 1.68 points on the DASS-21 Stress subscale at follow-up. This result is consistent with the findings of Planchuelo-Gómez et al. [6], who observed an increase of 2.75 points on the Stress subscale, with an average score of 6.51 on the first survey and 9.26 on the second. Similar to depression levels, stress levels were associated with negative affect and detachment, in line with the results reported in our previous study and the existing literature [32]. Lastly, our findings indicated that, within our sample (which ranged in age from 18–70 years), young age was associated with increased stress. This result is aligned with the result of our previous study, and may reflect the fact that the younger population has greater access to COVID-19 information through social media. A second explanation could pertain to the fact that younger persons, contrary to those in the elderly population, were more likely to have to adapt to new working or educational environments during the lockdown in order to maintain their daily activities. In the literature, young age has been found to be associated with increased stress, particularly with regard to education and career [33,34].

## 6. Strengths and Limitations

The Italian government was one of the first governments in Europe to implement extraordinary measures to prevent the spread of COVID-19, including recommended health measures and a nationwide lockdown. Most Italian citizens were caught unprepared, and, as shown, they responded with a range of distress symptoms. The present study was implemented during an advanced phase of the spread of COVID-19 in Italy, and it built on our prior research, conducted at the beginning of the lockdown period. For this reason, it represents an important contribution to our understanding of the psychological implications of the COVID-19 pandemic, emphasizing its impact on well-being in both the short and long term.

Nevertheless, the present study has some limitations, and should be interpreted with caution. First, given the peculiar and extraordinary nature of the emergency situation, the results may not generalize beyond Italy. Second, the sample was mainly comprised of females, employees, and students; greater analysis of an older population with different characteristics may have provided additional insight and a more comprehensive picture of the psychological situation of the general Italian population. Third, the survey measure was implemented via the Internet and relied on voluntary sampling and self-reported data. Future research should seek to compare the present study data with that collected using other methods (e.g., semi-structured interviews, qualitative approaches, etc.). Finally, given the recent resurgence of COVID-19 in the Italian territory [35], it would be useful to conduct a further study to identify any additional changes in the psychological well-being of Italian citizens at this time.

## 7. Conclusions

A recent systematic review [36] examined psychological factors in the general public during the COVID-19 pandemic and highlighted risk factors for mental health problems. Worryingly, most of the studies analyzed in the review reported a high prevalence of adverse psychiatric symptoms in their respective samples. The COVID-19 pandemic represents an unprecedented threat to mental health throughout the world, in high-, middle-, and low-income countries. Thus, in addition to seeking to flatten the curve of viral transmission, governments must also give priority to preventing mental disorders (e.g., major depressive disorder, post-traumatic stress disorder) [37]. Because the COVID-19 pandemic is associated with highly significant levels of psychological distress that, in many cases, would meet the threshold for clinical relevance, preventing and mitigating the adverse psychological impact of COVID-19 must be an international public health priority.

Overall, our results showed an increase in stress and depression over 2 months of lockdown in Italy. Negative affect and detachment were associated with higher levels of both depression and stress, especially for those whose levels were higher at the beginning of the lockdown period. A less resilient coping style and childlessness were associated with increased levels of depression, whereas young age was related to higher stress levels.

This epidemiological picture could facilitate the identification of persons at greater risk of suffering from psychological distress, which can impair functioning and lead to psychopathological consequences. For instance, taking advantage of the more widespread use of Web technologies, experts might utilize online assessments to evaluate depression, stress, personality features, and coping style, especially in younger Italians. Such a screening process could reach a high number of people at risk for mental disease in a short span of time, and thereby prevent the spread of psychopathologies in the coming years and promote compliance with the government-recommended health measures [38].

## Figures and Tables

**Table 1:** Descriptive Statistics of the Sample.

Characteristic	Group	*N* (%)
**Gender**	Female	329 (74.9%)
	Male	110 (25.1%)
**Age**	34.70 (13.15) 18–70	439 (100%)
***M* (*SD*)**		
**Min–Max**		
**Citizenship**	Italian	436 (99.3%)
	Foreign	3 (0.7%)
**Region of residence**	North	92 (21%)
	Center	246 (56%)
	South	101 (23%)
**Education**	Middle school diploma	11 (2.5%)
	High school diploma	139 (31.7%)
	Graduate	213 (48.5%)
	Postgraduate	76 (17.3%)
**Marital status**	Unmarried	273 (62.2%)
	Married	143 (32.6%)
	Separated/divorced	16 (3.6%)
	Widower	7 (1.6%)
**Occupation**	Employee	167 (62%)
	Freelancer	82 (18.7%)
	Unemployed	37 (8.4%)
	Student	151 (34.4%)
	Retired	2 (0.5%)
**Child(ren) in house**	No	316 (72%)
	Yes	123 (28%)
**Moved after the onset of the COVID-19 emergency**	No	395 (90%)
	Yes	13 (3%)
	Yes, to get closer to loved ones	31 (7%)
**Spending social distancing period with**	Family	320 (73.0%)
	Alone	53 (12.0%)
	Roommate(s)	41 (9.3%)
	Partner	25 (5.7%)
**Condition**	Must go to work	63 (14.4%)
	Can stay at home	376 (85.6%)
**Quarantine**	No	395 (90%)
	Yes, with family	33 (7.5%)
	Yes, alone	11 (2.5%)
**Number of times you leave your domicile each day**	0–1	404 (92%)
	2	23 (5.2%)
	2+	12 (2.7%)
**Reason for leaving domicile**	Key worker	64 (14.6%)
	Health reasons	21 (4.8%)
	Return home	23 (5.2%)
	State of need	331 (75.4%)
**Use of social media** **(hours)**	1–2	144 (32.8%)
	3–5	215 (49%)
	5–8	56 (12.8%)
	8–10	18 (4.1%)
	10+	6 (1.4%)
**Infected acquaintances**	No	340 (77.4%)
	Yes	99 (22.6%)
**Deaths among infected acquaintances**	No	423 (96.4%)
	Yes	16 (3.6%)
**Infected loved ones**	No	409 (93.2%)
	Yes	30 (6.8%)
**History of stressful situations**	No	265 (60.4%)
	Yes	174 (39.6%)
**History of medical problems**	No	311 (70.8%)
	Yes	128 (29.2%)
**Psychological support or psychotherapy**	No	314 (71.5%)
	Yes	125 (28.5%)
**Application of health-related measures**	From late February	149 (33.9%)
	From the first days of March	183 (41.7%)
	From the second week of March	102 (23.5%)
	From the third week of March	5 (1.1%)
**Social support from government**	Very sufficient	3 (0.7%)
	Quite enough	145 (33%)
	Enough	73 (16.6%)
	Not enough	164 (37.4%)
	Not enough at all	54 (12.3%)
**Reliability of government information**	Very reliable	100 (22.8%)
	Quite reliable	275 (62.6%)
	Unreliable	4 (0.9%)
	Not reliable at all	60 (13.7%)
**Detailed government information**	Very detailed	26 (5.9%)
	Quite detailed	226 (51.5%)
	Detailed	95 (21.6%)
	Not very detailed	80 (18.2%)
	Not detailed at all	12 (2.7%)
**Frequency of updates on COVID-19**	No	6 (1.4%)
	Yes, everyday	203 (46.2%)
	Yes, sometimes	61 (13.9%)
	Yes, many times per day	169 (38.5%)

**Table 2:** Difference Between the Initial and the Final Period of the Lockdown in Levels of Stress, Anxiety, and Depression, as Measured with the DASS-21.

DASS-21 Subscale	*M* (*SD*) Initial	*M* (*SD*) Final	*df*	*t*	*p*	Cohen’s *d*	95% CI for Cohen’s *d*	
							*Lower*	*Upper*
DASS-21 Depression	5.50 (4.88)	6.79 (5.40)	438	−6.505	2.120 × 10^−10^	−0.310	−0.406	−0.215
DASS-21 Anxiety	3.14 (3.95)	3.44 (4.14)	438	−1.752	0.080	−0.084	−0.177	0.010
DASS-21 Stress	7.69 (5.56)	9.37 (5.66)	438	−7.604	1.763 × 10^−13^	−0.363	−0.459	−0.266

**Table 3:** Multiple Linear Regression Model Predicting the DASS-21 Depression Subscale Score During the Final Period of the Lockdown.

Predictors	Δ*R*^2^	Unstandardized Coefficients (B)	S.E.	*t*	*p*	95% CI	
						*Lower Bound*	*Upper Bound*
DASS-21 Depression initial	0.460	0.584	0.044	13.374	2.177 × 10^−34^	0.498	0.670
PID-5-BF Negative Affect	0.033	0.202	0.073	2.769	0.006	0.059	0.345
PID-5-BF Detachment	0.013	0.255	0.082	3.116	0.002	0.094	0.415
BRCS	0.007	−0.176	0.069	−2.533	0.012	−0.312	−0.039
Having a child (yes)	0.006	−0.946	0.410	−2.304	0.022	−1.752	−0.139

*Note*: RMSE = 3.762. ANOVA *F*(5,433) = 93.553, *p* < 0.001. BRCS = Brief Resilient Coping Scale.

**Table 4:** Multiple Linear Regression Model Predicting the DASS-21 Stress Subscale Score During the Final Period of the Lockdown.

Predictors	Δ*R*^2^	Unstandardized Coefficients (B)	S.E.	*t*	*p*	95% CI	
						*Lower Bound*	*Upper Bound*
DASS-21 Stress initial	0.435	0.559	0.039	14.429	7.879 × 10^−39^	0.483	0.636
PID-5-BF Negative Affect	0.473	0.238	0.079	3.009	0.003	0.083	0.393
PID-5-BF Detachment	0.494	0.186	0.085	2.175	0.030	0.018	0.354
Age	0.488	−0.059	0.015	−3.811	1.585 × 10^−4^	−0.089	−0.028

*Note:* RMSE=4.043. ANOVA *F*_(4,434)_=105.823, *p* < 0.001.
